# A Study on the Correlation Between Driving Behavior and ECG Data in Driving Fatigue

**DOI:** 10.3390/s26113441

**Published:** 2026-05-29

**Authors:** Jiayou Wang, Chaoqun Zhang, Haocheng Xu, Peng He

**Affiliations:** 1 School of Mechanical Engineering, Xinjiang University, Urumqi 830017, China; 107552504270@stu.xju.edu.cn; 2 School of Traffic and Transportation Engineering, Xinjiang University, Urumqi 830017, China; 20252802215@stu.xju.edu.cn (C.Z.); 20252802218@stu.xju.edu.cn (H.X.); 3 Xinjiang Key Laboratory of Green Construction and Smart Traffic Control of Transportation Infrastructure, Xinjiang University, Urumqi 830017, China

**Keywords:** driving fatigue, driving behavior data, HRV, autonomic nervous system, fatigue detection model

## Abstract

Background: Fatigued driving is a key contributing factor to major traffic accidents. Existing detection technologies suffer from issues such as delayed identification, high error rates, and a lack of quantified causal relationships between physiological and behavioral indicators. This study aims to clarify the intrinsic relationship between electrophysiological and driving behavior data during the progression of driving fatigue. Methods: Four categories of driving behavior data and electrocardiographic (ECG) heart rate variability (HRV) indicators were selected as the study subjects. Based on a four-stage standardized simulated driving experiment ranging from wakefulness to severe fatigue, the correlations between indicators were quantified using Pearson correlation analysis, and a four-layer physiological–behavioral fusion fatigue assessment model was constructed. Results: Autonomic dysregulation is the intrinsic cause of abnormal driving behavior. The two exhibit a highly synchronized, stepwise progressive evolution pattern, with |r| ≥ 0.75 among core indicators. The accuracy of the constructed model exceeded 90% for all fatigue stages, reaching 97.8% for severe fatigue detection, with a response time of ≤0.5 s. Conclusions: This model effectively addresses the limitations of single-monitoring technologies and provides theoretical support and technical guidance for multimodal identification and graded early warning of driving fatigue.

## 1. Introduction

Fatigued driving is a major contributor to traffic accidents, often leading to severe property damage and casualties. Relevant surveys indicate that accidents attributed to fatigued driving account for approximately 20% of all traffic incidents and over 40% of fatal crashes [1]. Driving fatigue impairs reaction times, decision-making, and attention allocation, increasing the likelihood of operational errors and reducing the driver’s ability to respond promptly to unexpected road events [2].

Electrocardiogram (ECG) data, as a direct reflection of autonomic nervous system activity, has become a reliable physiological marker for driving fatigue detection. Meanwhile, driving behavior data can intuitively characterize a driver’s real-time risk state. However, existing studies have not fully clarified the intrinsic quantitative correlation between the dynamic evolution of ECG indicators and abnormal changes in driving behavior during fatigue progression. Exploring the synchronous coupling relationship between these two modalities can provide a new theoretical basis for overcoming the limitations of single-modal fatigue detection technology.

Scholars both domestically and internationally have conducted extensive research on driver fatigue detection methods across multiple dimensions, including driving behavior [3], physiological signals [4,5,6], and image information [7]. In addition, numerous related studies have laid a foundation for the development of driver fatigue detection technology [8,9,10,11,12,13,14,15,16,17,18,19,20,21,22,23,24,25,26,27,28,29,30]. Current mainstream driver fatigue detection technologies are primarily divided into two categories: those relying on driving behavior data and those relying on physiological signals. However, driving behavior-based detection methods are prone to recognition lag, making it impossible to capture the subtle characteristics of early-stage fatigue during driving or to trace the dynamic evolution of early fatigue; conversely, detection methods relying on physiological signals cannot directly reflect the actual driving risk levels resulting from the driver’s operations. Additionally, existing research still faces challenges such as insufficient rationality in the allocation of indicator weights, ambiguous definitions of fatigue grading thresholds, and a lack of quantifiable causal relationships between physiological indicators and behavioral data.

The main innovations of this paper are threefold: first, we clarified the quantitative correlation between progressive imbalances in the autonomic nervous system and abnormal driving behaviors, establishing a clear causal link between the two; second, we constructed a four-tiered physiological–behavioral fusion model to achieve bidirectional validation of physiological indicators and behavioral characteristics, thereby enabling the precise classification of driving fatigue; third, we established quantifiable fatigue classification thresholds with practical engineering value, providing data support for the implementation of relevant detection technologies and offering a reference framework for real-world engineering applications.

## 2. Materials and Methods

### 2.1. Experimental Design and Data Collection

The baseline data for this study were obtained from standardized driving fatigue simulation experiments. The experimental paradigm combined Sudoku-induced cognitive fatigue with prolonged continuous driving, during which driving behavior data and electrocardiographic (ECG) data were collected simultaneously. The sampling frequency of driving behavior data was 50 Hz; ECG data were collected using an Equivital EQ 02+ LifeMonitor chest strap sensor (Equital S.r.l., Florence, Italy). Raw ECG signals were recorded at 256 Hz and exported as time-series data. RR intervals were automatically calculated from the ECG Lead signals using a custom Python3.10 script, and further heart rate variability (HRV) analysis (time-domain, frequency-domain, and nonlinear parameters) was performed using Kubios HRV Scientific software (Kubios Oy, Kuopio, Finland, version 3.5.0). 

A total of 15 healthy volunteers were recruited for this study, including 11 males and 4 females. All participants held a valid Class C1 driver’s license, had at least one year of driving experience, and reported no chronic or underlying medical conditions. To minimize the influence of confounding factors, participants were instructed to maintain sufficient sleep before the experiment and abstain from stimulants such as alcohol and caffeine. The driving simulation scenario was set on an urban expressway without unexpected events. The experimental environment was kept consistent throughout the test: temperature 23–25 °C, humidity 50–60%, and no strong light or noise interference.

Although 15 participants is a commonly used sample size in preliminary simulated driving studies, the limited sample may restrict the statistical robustness and generalization ability of the conclusions. This is a major limitation of this study.

The experiment employed a paradigm combining cognitive load and continuous driving to induce progressive driver fatigue: participants first completed a 15 min driving simulation to collect baseline data under alert conditions; subsequently, they alternated between 30 min Sudoku tasks and 15 min driving sessions to gradually induce mild, moderate, and severe fatigue states. To improve the reliability of fatigue state classification, multi-dimensional cross-validation was performed using Karolinska Sleepiness Scale (KSS) scale scores, driving behavior characteristics, and ECG-HRV indicators throughout the experiment. Throughout the experiment, cross-validation was performed using scale scores, driving behavior characteristics, and physiological indicators to ensure accurate classification of fatigue stages. The driver fatigue classification criteria based on KSS scores are shown in Table 1.

### 2.2. Key Research Indicators

#### 2.2.1. Key Driving Behavior Metrics

We have selected vehicle speed, throttle position, steering angle, and brake pedal position as the four core dimensions for analyzing driving behavior. From each dimension, we extract quantitative metrics such as fluctuation amplitude, operational stability, vibration density, self-centering/return-to-center capability, and peak position to comprehensively reflect the behavioral changes throughout the entire process—from precise control to loss of control—and provide reliable behavioral feature data for the model’s input layer. The formulas for the core quantitative metrics are as follows:

(1) Speed fluctuation range:

(1) $$\Delta v=max\left(v\right)-min\left(v\right)$$

In the formula, $max\left(v\right)$ represents the maximum vehicle speed (m/s) during the monitoring period; $min\left(v\right)$ represents the minimum vehicle speed (m/s) during the monitoring period.

(2) Operational stability:

(2) $$S=1-\frac{\sigma }{v_{avg}}$$

In the formula, σ represents the standard deviation of vehicle speed during the monitoring period (m/s); $v_{avg}$ represents the average vehicle speed during the monitoring period (m/s); S ∈ [0, 1], where a value closer to 1 indicates better stability in the driver’s speed control.

(3) Vibration density:

(3) $$D=\frac{N}{L}$$

In the formula, N represents the number of jolts caused by driving maneuvers during the monitoring period; L represents the distance traveled by the vehicle during the monitoring period (100 m). The jolt density provides a clear indication of the smoothness of driving maneuvers.

The characteristics of handling metrics at different levels of driver fatigue are shown in Table 2.

#### 2.2.2. Key Electrophysiological Parameters

With heart rate variability (HRV) as the primary focus, this study selects key electrophysiological indicators from five dimensions—basic physiology, the autonomic nervous system, the time domain, the frequency domain, and nonlinearity—to precisely reveal the core evolutionary patterns of autonomic nervous system dysregulation during the progression of fatigue, thereby providing scientific physiological data for the model’s input layer. Core indicators include mean heart rate, stress index, parasympathetic nervous system (PNS) index, sympathetic nervous system (SNS) index, standard deviation of normal RR intervals (SDNN), proportion of adjacent RR interval differences > 50 ms (pNN50), sample entropy (SampEn), and heart rate variability ratio (SD2/SD1). The calculation formulas for these core quantitative indicators are as follows:

(1) Average heart rate:

(4) $$HR_{avg}=\frac{60}{RR_{avg}}$$

In the equation, $RR_{avg}$ represents the average interval between adjacent RR intervals during the monitoring period (s); $HR_{avg}$ represents the average heart rate, measured in beats per minute (bpm).

(2) Standard deviation of the normal RR interval (SDNN):

(5) $$SDNN=\sqrt{\frac{1}{n-1}\sum_{i=1}^{n}{\left(RR_{i}-RR_{avg}\right)}^{2}}$$

In the equation, n represents the number of normal RR intervals during the monitoring period; $RR_{i}$ represents the duration of the i-th normal RR interval (ms); SDNN is a key indicator in HRV time-domain analysis that reflects the overall level of heart rate variability.

(3) pNN50:

(6) $$pNN50=\frac{Num(|RR_{i+1}-RR_{i}|>50\;ms)}{n-1}\times 100\%$$

In this equation, Num(·) represents the number of adjacent RR intervals with a difference greater than 50 ms; pNN50 reflects the characteristics of short-term heart rate variability and is highly correlated with parasympathetic activity.

(4) Sample entropy (SampEn):

(7) $$SampEn=-ln(\frac{C_{m}^{d}}{C_{m+1}^{d}})$$

In the equation, $C_{m}^{d}$ represents the proportion of similar vectors in an m-dimensional vector; d is the similarity threshold, which is set to 0.2 × SDNN in this study; SampEn is a nonlinear analysis metric that reflects the complexity of heart rate time series, with lower values indicating lower complexity in heart rate variability.

(5) Heart rate variability ratio (HRVR):

(8) $$HRVR=\frac{SD2}{SD1}$$

In this equation, SD1 represents the standard deviation of the short axis of the HRV scatter plot, reflecting short-term heart rate variability; SD2 represents the standard deviation of the long axis of the HRV scatter plot, reflecting long-term heart rate variability; HRVR reflects the self-similarity of heart rate variability and serves as an important nonlinear indicator for fatigue assessment. The detailed behavioral indicators of each fatigue phase are shown in Table 3.

### 2.3. Correlation Analysis Methods

This study uses Pearson’s correlation coefficient to perform a systematic correlation analysis on key quantitative indicators derived from driving behavior data and ECG-based HRV data, quantifying the strength of the association between the two modalities and providing empirical support for indicator weight assignment in the fusion model. The formula for Pearson’s correlation coefficient is as follows:

(9) $$r=\frac{\sum_{i=1}^{n}\left(x_{i}-\bar{x}\right)\left(y_{i}-\bar{y}\right)}{\sqrt{\sum_{i=1}^{n}{\left(x_{i}-\bar{x}\right)}^{2}}\cdot \sqrt{\sum_{i=1}^{n}{\left(y_{i}-\bar{y}\right)}^{2}}}$$

In the equation, r is the Pearson correlation coefficient, where r ∈ [−1, 1]; $x_{i}$ is the i-th ECG parameter value, and x is the mean value of the ECG parameters; $y_{i}$ is the i-th driving behavior parameter value, and y is the mean value of the driving behavior parameters; n is the sample size. When r > 0, the correlation is positive; when r < 0, the correlation is negative; when |r| ≥ 0.8, the correlation is strong; and when 0.5 ≤ |r| < 0.8, the correlation is moderate.

### 2.4. Model Development and Validation Methods

This study constructs a four-layer physiological–behavioral fusion fatigue assessment model consisting of an input layer, a feature fusion layer, a decision layer, and an output layer. Data preprocessing is implemented using Min–Max normalization, feature fusion is realized via a weighted fusion algorithm, and fatigue classification is determined using bidirectional validation rules.

The dataset was divided into training and validation sets at an 8:2 ratio: 80% of the data were used to adjust model weights, optimize classification thresholds, and fine-tune other parameters, while the remaining 20% were applied to validate the classification accuracy of the model. During training, Mean Squared Error (MSE) was adopted as the loss function for the iterative optimization of model parameters. The formula for the loss function is as follows:

(10) $$MSE=\frac{1}{n}\sum_{i=1}^{n}(F_{i}-{\hat{F}}_{i}{)}^{2}$$

In this equation, $F_{i}$ represents the actual fused eigenvalue of the i-th sample; ${\hat{F}}_{i}$ represents the fused eigenvalue predicted by the model; n represents the number of training samples; the smaller the MSE, the better the model fits the data.

Using classification accuracy as the core metric for evaluating model effectiveness, the calculation formula is as follows:

(11) $$Accuracy=\frac{N_{corr}}{N_{total}}\times 100\%$$

In the formula, $N_{corr}$ represents the number of samples correctly classified by the model, and $N_{total}$ represents the total number of samples in the validation set.

This study was conducted in accordance with the Declaration of Helsinki and approved by the Institutional Review Board of Xinjiang University.

### 2.5. Technical Principle of the Fusion Model

The four-layer physiological–behavioral fusion driving fatigue assessment model is a multimodal classification framework established based on Pearson correlation quantification and adaptive weighted feature fusion. This model regards autonomic nervous system dysregulation (characterized by ECG-HRV indicators) as the intrinsic physiological mechanism of driving fatigue, and abnormal fluctuations in driving behavior as the external operational phenotype. Through feature-level weighted fusion and decision-level threshold mapping, the model enables bidirectional corroboration and hierarchical quantitative identification of driver fatigue states.

Min–Max normalization is applied to remove dimensional and magnitude discrepancies among heterogeneous physiological and behavioral indicators, with all features scaled to the range [0, 1] to ensure comparability and numerical stability in multi-source data fusion. Indicator weights are adaptively assigned according to the Pearson correlation between each feature and fatigue severity, and key indicators with |r| ≥ 0.75 are highlighted to enhance sensitivity to fatigue evolution. A dual-modal cross-validation mechanism is introduced to reduce misclassification caused by signal noise, individual differences, or transient disturbances. Hierarchical quantitative recognition is achieved using the fused feature F and three optimized thresholds (0.25, 0.50, 0.75), enabling continuous and reliable detection across the full progression from alertness to severe fatigue.

By integrating the early predictive capability of physiological signals and the risk-oriented representation of driving behavior, the model alleviates the drawbacks of conventional single-modal detection methods, including recognition lag, low accuracy, and limited information dimensionality. Accordingly, it provides reliable theoretical and technical support for the real-time, high-precision monitoring and graded early warning of driving fatigue.

## 3. Results

### 3.1. Synchronous Evolutionary Characteristics of ECG and Driving Behavior Data Across the Four Stages of Driver Fatigue

Based on a four-stage classification system—alert, mild fatigue, moderate fatigue, and severe fatigue—this study systematically analyzes the synchronous evolution of core electrophysiological and driving behavior data throughout the full progression of driver fatigue. It reveals the stepwise, progressively coupled characteristics between autonomic nervous system function and driving control ability, providing a key foundation for the quantitative analysis of their relationship.

#### 3.1.1. Awake State: Physiological Homeostasis, Precise Behavior

The driver’s autonomic nervous system is in a state of dynamic equilibrium, with a PNS index of approximately 7.04 and an SNS index of approximately 4.33; there are no abnormalities in sympathetic or parasympathetic nerve activation; HRV indicators remained at baseline levels, with SDNN ≈ 248.2 ms, pNN50 ≥ 61.31%, LF/HF ≈ 0.365, and the stress index ≤ 3.8, indicating a low level of cardiac load.

**Figure 1:**
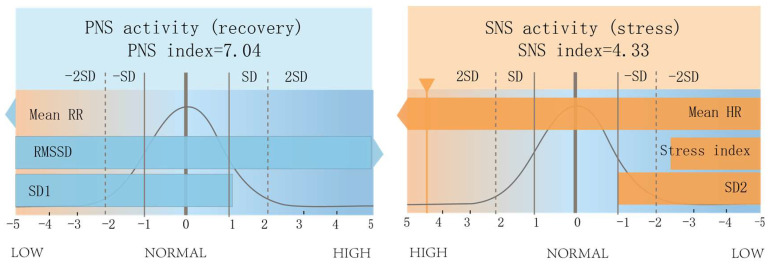
Characteristics of autonomic balance and heart rate variability (HRV) indices during the waking driving phase.

The driver’s driving behavior exhibits characteristics of active and precise control: average vehicle speed is 14.16 m/s, with a fluctuation range of ≤±3 m/s, and the speed curve is smooth with no oscillations; average throttle opening is 0.4–0.5, with 100% operational stability, and the throttle returns quickly after full-throttle operation; the steering wheel angle remained stable at ±0.35 rad, with a jitter density of 0 events per 100 m and a return-to-center response time ≤ 50 ms; the average brake pedal travel was ≈0.03, with a maximum brake travel of ≤0.4, and the pedal returned to its original position immediately after braking, indicating extremely low driving risk, see Figure 1.

#### 3.1.2. Mild Fatigue: Physiological Adaptation; Initial Decline in Behavioral Accuracy

The driver’s autonomic nervous system enters a brief state of sub-equilibrium, with no significant physiological signs of fatigue: the PNS index is ≥9.87, an increase of ≥40.2% compared to the awake state; the SNS index is ≈4.24, a decrease of ≤2.1% compared to the awake state, indicating brief activation of the parasympathetic nervous system. HRV metrics reached peak levels: SDNN ≥ 341.1 ms, an increase of ≥37.4% compared to the awake state; pNN50 remained at 61.31%; total power in the frequency domain ≥ 548,612 ms^2^, an increase of ≥194.5% compared to the awake state; stress index ≤ 2.6, a decrease of ≤31.6% compared to the awake state.

**Figure 2:**
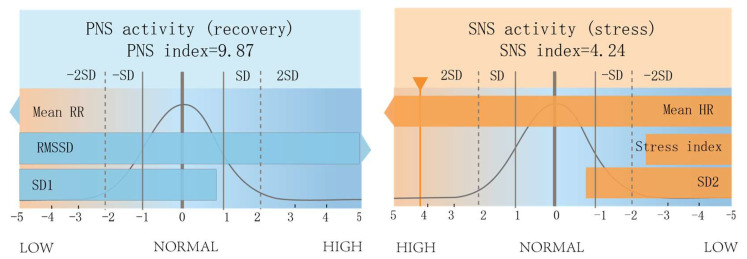
Characteristics of autonomic stress adaptation and heart rate variability indices during the mild fatigue phase.

Driving behavior exhibited reduced precision in fine control and a pseudo-stable pattern: the average vehicle speed was 12.15 m/s, with a fluctuation range of ±3.5 m/s, and the speed curve displayed localized sawtooth-like fluctuations; average throttle opening was 0.2–0.3, with operational stability dropping to 80%, and throttle operation exhibiting high-frequency, small-amplitude oscillations; steering wheel angle was ±0.42 rad, an increase of ≥20% compared to the awake state, with oscillation density ≤ 2 times/100 m, and steering wheel return response time ≤ 60 ms; maximum brake pedal travel ≤ 0.55, an increase of ≥37.5% compared to the awake state; brake pedal return response time ≤ 60 ms; driving risk is low, but clear signs of incipient fatigue have already appeared, see Figure 2.

#### 3.1.3. Moderate Fatigue: Physiological Imbalance, Disruption of Behavioral Feedback

The driver’s autonomic nervous system regulation has entered a critical turning point, with the sympathetic-parasympathetic balance disrupted: the SNS index is ≥7.62, representing an increase of ≥76% compared to the awake state; the PNS index ≤ 5.92, a decrease of ≥15.9% compared to the awake state. HRV metrics show a sharp decline, with SDNN ≈ 260.6 ms, a decrease of ≥23.6% from peak levels; pNN50 ≤ 47.0%, a decrease of ≥23.3% from peak levels; HF percentage ≤ 56.62%, a decrease of ≥20.2% compared to the awake state; the LF/HF ratio continued to rise, with the stress index ≥ 4.0, indicating an explosive increase in cardiac load.

**Figure 3:**
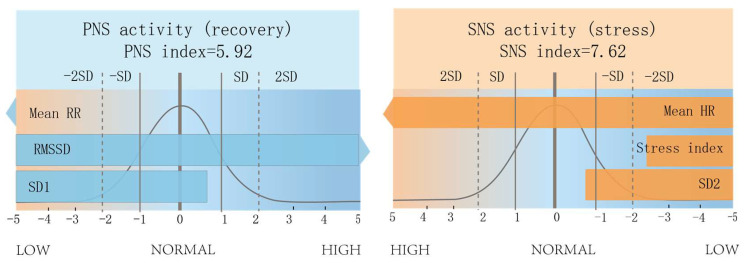
Characteristics of autonomic imbalance and reduced heart rate variability indices during the moderate fatigue phase.

The driver’s driving behavior exhibited a disruption in the visual-central-motor feedback loop, resulting in a passive error-correction mode: the average vehicle speed was 11.18 m/s, with a fluctuation range of ±8.10 m/s; the speed curve displayed high-frequency jagged patterns, frequently spiking and then dropping; average throttle opening was 0.3–0.4, with control stability dropping to 50%; operations alternated irregularly between full throttle and zero throttle; the oscillation frequency of both the steering wheel and brakes was ≥8 times per 100 m, an increase of ≥300% compared to the mild fatigue stage; The steering wheel return response time is 120–150 ms, and the maximum brake pedal travel is ≥0.7, with driving risk rising to a medium-high level, see Figure 3.

#### 3.1.4. Severe Fatigue: Physical Exhaustion, Complete Loss of Behavioral Control

The driver’s autonomic nervous system is on the verge of decompensatory collapse and is in a state of excessive stress: the PNS index is ≤2.70, a decrease of ≥61.6% compared to the awake state; the SNS index is ≥9.61, an increase of ≥122% compared to the awake state; the PNS percentage and HF power percentage are falsely elevated. HRV metrics are nearly absent, with SDNN ≤ 160.0 ms, a decrease of ≥53.1% from peak levels; pNN50 ≤ 36.68%, a decrease of ≥40.2% from peak levels; total power in the frequency domain ≤ 29,983 ms^2^, a decrease of ≥94.5% from peak levels; nonlinear indices exhibited abnormal characteristics: SampEn ≤ 0.253, a decrease of ≥73.5% compared to the awake state; HRVR < 1, indicating a complete loss of self-similarity in heart rate fluctuations; and the stress index ≥ 7.8, an increase of ≥105% compared to the awake state, with cardiac load reaching its maximum.

**Figure 4:**
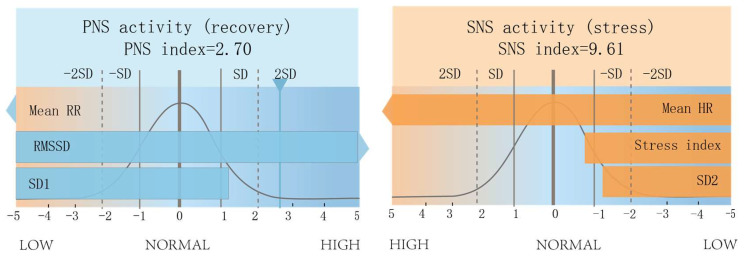
Characteristics of autonomic nervous system suppression and decreased heart rate variability indices during the stage of severe fatigue.

The driver’s driving behavior exhibited complete failure of central control, entering an unconscious loss-of-control mode: average vehicle speed was 20–30 m/s, later even exceeding 40 m/s, with a fluctuation amplitude of ≥±15 m/s, exhibiting extreme oscillations and uncontrolled acceleration; the average throttle opening is 0.5–0.6, with operational stability ≤ 10%; sustained full throttle (opening = 1.0 for ≥500 m) occurs without any reduction; the oscillation frequency of both the steering wheel and brakes is ≥10 times per 100 m; the steering wheel return response time is ≥200 m, with return fluctuations ≥ 8 times; the maximum brake pedal travel is ≤0.22, a decrease of ≥68.6% compared to the moderate fatigue stage; brake pedal return fluctuations are ≥5 times. At this point, the driver’s consciousness is impaired, driving operations are completely out of control, driving risk reaches an extremely high level, and the likelihood of causing a serious traffic accident is extremely high, see Figure 4.

### 3.2. Quantitative Relationships Among Key Performance Indicators

According to Pearson correlation analysis, the relationship between driving behavior and key ECG HRV indicators is primarily reflected in three aspects:

Negative correlation: ECG physiological indicators such as the PNS index, SDNN, RMSSD, and SampEn show a significant negative correlation (r ≤ −0.75) with driving behavior indicators including oscillation density, fluctuation amplitude, and steering/braking peaks. In other words, the lower the physiological indicator values, the higher the degree of driving behavior abnormality.

Positive correlation: ECG physiological indicators such as the SNS index, average heart rate, and stress index show a significant positive correlation (r ≥ 0.78) with driving behavior indicators such as oscillation density and fluctuation amplitude. In other words, the higher the physiological indicator values, the greater the degree of driving behavior abnormality.

Threshold correspondence: The core early-warning thresholds for severe fatigue derived from electrocardiographic indicators are fully consistent with the core validation thresholds from driving behavior indicators, forming mutually corroborative diagnostic criteria and providing a key foundation for threshold setting in the fusion model.

### 3.3. Results of the Development of a Physiological–Behavioral Integrated Model for Assessing Driver Fatigue

#### 3.3.1. Overall Model Architecture

**Figure 5:**
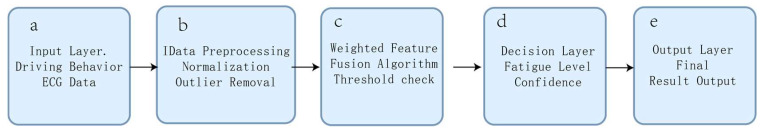
Architecture diagram of the physiological–behavioral integrated fatigue detection model. Detailed architecture of the four-layer physiological–behavioral fusion fatigue assessment model. (**a**) Input layer: ECG-HRV and driving behavior data input; (**b**) Preprocessing layer: outlier removal and Min–Max normalization; (**c**) feature fusion layer: adaptive weighted fusion based on Pearson correlation; (**d**) decision layer: dual-modal cross-validation and fatigue classification; (**e**) output layer: fatigue level, confidence and risk level output.

The physiological–behavioral integrated fatigue assessment model developed in this study employs a four-layer structured design comprising an input layer, a feature fusion layer, a decision layer, and an output layer. Each layer has a clearly defined function and is easy to implement, allowing the model to be directly integrated into existing driver fatigue monitoring systems. The overall architecture of the model is shown in Figure 5.

**Input layer**

The model input layer is divided into two modules that separately process physiological and behavioral features. These modules synchronously collect two types of core data, perform standardization to eliminate dimensional differences, and support metric comparison across dimensions. The physiological feature module integrates six core HRV metrics covering four major dimensions: autonomic nervous system, time domain, nonlinearity, and basic physiology. The behavioral feature module incorporates five key driving behavior metrics covering four control dimensions: vehicle speed, throttle, steering wheel, and brakes.

All raw input data undergoes Min–Max standardization, mapping the data to the [0, 1] interval. The calculation formula is as follows:

(12) $$x_{norm}=\frac{x-x_{min}}{x_{max}-x_{min}}$$

In the formula, $x_{norm}$ represents the normalized data, where $x_{norm}$ ∈ [0, 1]; x represents the raw indicator data; $x_{min}$ is the minimum value of the indicator across all samples; and $x_{max}$ is the maximum value of the indicator across all samples.

**Feature Fusion Layer**

The feature fusion layer constitutes the core and critical component of the model. It adopts a weighted fusion algorithm that integrates the quantified correlations between driving behavior and electrocardiographic (ECG) indicators. Indicator weights are assigned following the principle that “the higher the fatigue level, the greater the weight of the core indicators”. Specifically, the four core indicators for the severe fatigue stage are assigned a weight of 0.15, the three core indicators for the mild fatigue stage are assigned 0.12, and the weights of the remaining indicators are set to 0.08–0.10. All indicator weights satisfy the normalization condition:

(13) $$\sum_{i=1}^{k}w_{i}=1$$

In the formula, $w_{i}$ represents the weight of the i-th indicator; k represents the total number of fusion indicators, which is 11 in this study.

Using a weighted fusion algorithm, the standardized physiological and behavioral features are converted into a unified fusion feature vector, calculated as follows:

(14) $$F=\sum_{i=1}^{k}w_{i}\cdot x_{norm,i}$$

In the equation, F represents the fusion feature vector value, where F∈[0,1]; $x_{norm,i}$ represents the normalized data for the i-th indicator; the higher the fusion feature vector value, the more severe the driver’s fatigue.

**Decision layer**

The decision layer constructs its decision logic based on the values of the fused feature vectors and the thresholds for the four-stage classification of driver fatigue. It incorporates a “two-way verification” rule to mitigate model misclassification. The classification thresholds for each fatigue stage are determined through model training and optimization to improve the model’s classification accuracy. The decision logic formula is as follows:

(15) $$Level=\left\{\begin{array}{ll} Sober, & F\leq 0.25\\ Mild\;fatigue, & 0.25<F\leq 0.50\\ Moderate\;fatigue, & 0.5<F\leq 0.75\\ Severe\;fatigue, & F>0.75 \end{array}\right.$$

In the formula, “Level” represents the fatigue classification result; 0.25, 0.50, and 0.75 are the fatigue classification thresholds determined after model training and optimization.

Cross-validation rule: A driver is classified into a specific fatigue level only when both physiological and behavioral data reach the thresholds corresponding to that fatigue stage; if only one type of data meets the threshold, the classification is lowered by one level. This approach mitigates model misclassification caused by data errors.

**Output layer**

The core function of the output layer is to generate real-time fatigue classification results (alert, mild, moderate, severe), identify key abnormal indicators that trigger alerts (such as elevated PNS indices and increased speed variability), and assign corresponding risk levels, thereby providing clear technical support for driver fatigue warnings.

#### 3.3.2. Model Validation Results

**Figure 6:**
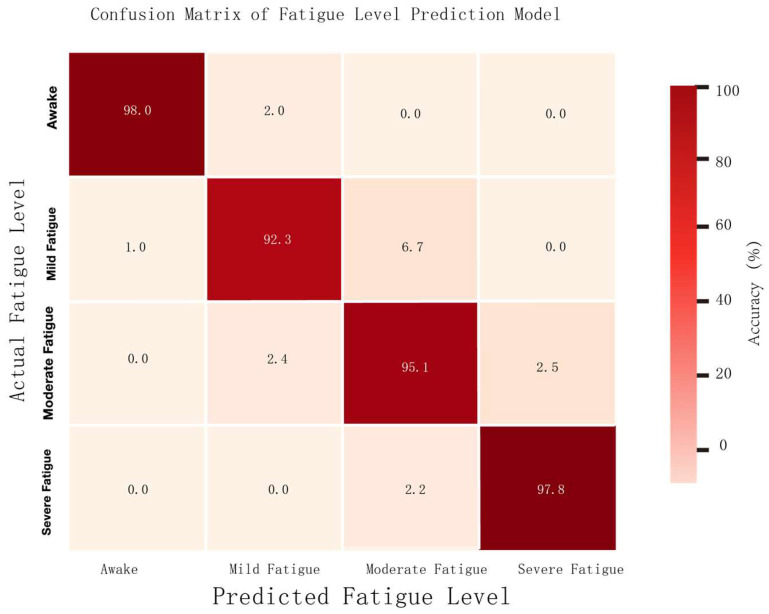
Physiological–behavioral integrated driving fatigue detection model confusion matrix.

The model validation results indicate that the model achieves an accuracy rate exceeding 90% for the classification of all fatigue stages, with an accuracy rate of 92.3% for mild fatigue, 95.1% for moderate fatigue, and 97.8% for severe fatigue. The overall misclassification rate is less than 8%, which fully demonstrates the model’s effectiveness and reliability; additionally, the model’s response time is ≤0.5 s, meeting the real-time and rapid detection requirements for driver fatigue monitoring in engineering applications. The model’s confusion matrix is shown in Figure 6.

### 3.4. Thresholds for Classifying Driver Fatigue

Based on the results of quantitative correlation analysis between driving behavior and electrophysiological data, as well as the training of the fusion model, a two-way verification and grading system for fatigue detection and assessment has been established, wherein “electrophysiological indicators serve as the internal basis for judgment, driving behavior indicators serve as the external basis for verification, and the fusion model serves as the core vehicle.” This system clarifies the core quantitative thresholds for the four stages of driving fatigue, ensuring the accuracy and practicality of fatigue level assessments. The core determination thresholds for each fatigue stage and the model output results are shown in Table 4.

## 4. Discussion

Based on the full cycle of onset and progression of driving fatigue, this study systematically reveals the synchronous evolutionary patterns between electrocardiographic (ECG) and behavioral indicators. It confirms that the gradual imbalance of autonomic regulation acts as the intrinsic physiological cause of progressive abnormalities in driving behavior, clarifies the causal logic that “physiology determines behavior, and behavior reflects physiology,” and addresses the research gap of insufficient quantitative causal relationships between these two types of indicators.

Compared with the studies of Zeng et al. [7] and Zambrano et al. [8] published in Sensors, the proposed fusion model improves the severe fatigue detection accuracy to 97.8% and reduces the response time to ≤0.5 s, showing better real-time performance and higher detection accuracy than single-modal methods.

Compared with existing single-dimensional studies on driving fatigue detection, the physiology–behavior fusion model proposed in this study effectively alleviates typical technical limitations—including recognition lag in behavior-based detection and the inability of physiology-based methods to directly reflect driving risks—via bidirectional validation rules. The model achieves 97.8% accuracy in severe fatigue identification with a response time ≤ 0.5 s, significantly outperforming most conventional single-modal detection models. It also exhibits an extremely low false positive rate, satisfying the engineering requirements for real-time monitoring in in-vehicle applications.

The four-stage fatigue classification thresholds established in this study resolve the issues of ambiguous threshold definitions and inconsistent standards in existing research, providing a standardized foundation for multimodal fatigue recognition and early warning systems. Furthermore, this study identified activation characteristics of the parasympathetic nervous system during the mild fatigue stage, offering key physiological biomarkers for the early identification and intervention of driver fatigue. This has significant practical implications for reducing the incidence of traffic accidents caused by fatigued driving.

This study still has certain limitations: First, the experimental sample size is only 15 participants, which limits the statistical robustness and generalization ability of the conclusions. Second, the experiment was carried out in a simulated driving environment with short-time driving segments and only Sudoku-induced cognitive load, which is quite different from real road scenarios and may not fully induce real moderate and severe fatigue states. Third, the analysis did not account for the influence of demographic attributes such as age, gender, and driving experience. Future research will further expand the sample size, conduct real-vehicle road tests, optimize the model’s adaptability under complex road conditions, and promote the practical application of the model in fatigue monitoring systems for intelligent connected vehicles.

## Figures and Tables

**Table 1:** Driver fatigue state classification standard based on KSS score.

Fatigue Phase	KSS Score Range	Key Features
awake	≤3 points	The driver remained focused and demonstrated precise control, with no fatigue-related physiological or behavioral abnormalities.
Mild fatigue	4–5 points	A slight decline in fine motor control accuracy and a brief state of autonomic imbalance indicate the early stages of fatigue.
Moderate fatigue	6–7 points	Significant autonomic nervous system imbalance; driving operates in a passive “deviate-then-correct” mode, indicating a stage of significant fatigue.
Severe fatigue	≥8 points	The autonomic nervous system is on the verge of decompensation, posing a risk of loss of control during driving; this constitutes a dangerous stage of fatigue.

**Table 2:** Characteristics of control indicators under different degrees of driving fatigue.

Control Dimensions	Analytical Indicators	Sober	Mild Fatigue	Moderate Fatigue	Severe Fatigue
Speed	Average speed (m/s)	14.00–16.00	12.00–15.00	11.00–18.00	20.00–30.00
	range of fluctuation (±m/s)	≤±3.00	±3.00–5.00	±8.00–10.00	≥±15.00
Steering wheel angle	Steady-state oscillatory density (times per 100 m)	0	≤2.00	≥8.00	≥10.00
	Steering angle (rad)	±0.35	±0.42	No fixed value; slight fluctuation	No fixed value; scattered randomly
Throttle opening	Average opening	0.40–0.50	0.20–0.30	0.30–0.40	0.50–0.60
Brake pedal travel	Maximum brake opening	0.40	0.50	0.70	0.20
	Steady-state oscillation density (times per 100 m)	0	≤2.00	≥8.00	≥10.00

Note: All values represent the full range of observed data across participants. The relationship between throttle opening and speed is influenced by individual driving habits and the experimental instruction to maintain a safe driving speed. Analysis in this study focuses primarily on trends in control stability (e.g., speed fluctuation, steering angle variation), rather than absolute speed values.

**Table 3:** Physiological and heart rate variability indicators of different degrees of driving fatigue.

Physiological Dimension	Key Metrics	Sober	Mild Fatigue	Moderate Fatigue	Severe Fatigue
Basic Physiology	Stress Index	3.80	2.60	4.00	7.80
Autonomic nervous system	PNS Index	7.04	9.87	5.92	2.70
	SNS Index	4.33	4.24	7.62	9.61
Time-domain analysis	SDNN (ms)	248.20	341.10	260.60	160.00
	pNN50 (%)	61.31	61.31	47.24	36.68
Frequency Domain Analysis	Total power (ms^2^)	186,300.00	548,612.00	108,142.00	29,983.00
	LF/HFratio	0.37	0.32	0.29	0.21
Nonlinear Analysis	SampEn	0.95	0.65	0.61	0.25
	HRVR	1.04	1.20	1.28	0.99

**Table 4:** Core judgment limits and model output of four stages of driving fatigue.

Fatigue Phase	Core Electrophysiological Thresholds	Thresholds for Determining Core Driving Behaviors	Fusion Eigenvalue Range
awake	Stress Index ≤ 3.8; PNS ≈ 7.04, SNS ≈ 4.33; SDNN ≈ 248.20 ms, SampEn ≥ 0.95	Speed fluctuations ≤ ±3 m/s, Operational stability ≥ 95%, Vibration density = 0 per 100 m	F ≤ 0.25
Mild fatigue	Stress Index ≤ 2.60; PNS ≥ 9.87; SDNN ≥ 341.10 ms, SampEn ≈ 0.65	Speed fluctuations ±3 to ±5 m/s, Operational stability 80–90%, Vibration density ≤ 2 per 100 m	0.25 < F ≤ 0.5
Moderate fatigue	Stress Index ≥ 4.00; PNS ≤ 5.92, SNS ≥ 7.62; SDNN ≈ 260.60 ms	Speed fluctuations ±8 to ±10 m/s, Operational stability 50–60%, Vibration density ≥ 8 per 100 m	0.5 < F ≤ 0.75
Severe fatigue	Stress Index ≥ 7.8; PNS ≤ 2.70, SNS ≥ 9.61; SDNN ≤ 160.00 ms, SampEn ≤ 0.25	Speed fluctuations ≥ ±15 m/s, Operational stability ≤ 10%, Vibration density ≥ 10 per 100 m	F > 0.75

## Data Availability

The data presented in this study are available on request from the corresponding author due to the privacy of research participants.

## Abbreviations

The following abbreviations are used in this manuscript:

Abbreviation	Full Name
HRV	Heart Rate Variability
KSS	Karolinska Sleepiness Scale
PNS	Parasympathetic Nervous System Index
SNS	Sympathetic Nervous System Index
SDNN	Standard Deviation of Normal NN Intervals
pNN50	Percentage of NN Intervals Differing by More Than 50 ms
SampEn	Sample Entropy
MSE	Mean Squared Error
HRVR	Heart Rate Variability Ratio (SD2/SD1)
